# Anchoring of esophageal stents with through-the-scope suturing devices: Preclinical proof of concept and first clinical cases.

**DOI:** 10.1055/a-2760-6455

**Published:** 2025-12-19

**Authors:** Gabriel Marcellier, Birane Beye, Nathaniel Edery, Alain Berson, Benedicte Jais, Paul Rivallin, Frédéric Prat

**Affiliations:** 1 55100 Endoscopy Unit, Beaujon Hospital, Clichy, France; 2 Gastroenterology, Orleans Hospital, Orleans, France; 3 55100 Pancreatology, Beaujon Hospital, Clichy, France

**Keywords:** Endoscopy Upper GI Tract, Benign strictures, Dilation, injection, stenting, Malignant strictures

## Abstract

**Background and study aims:**

Esophageal refractory benign strictures are challenging to manage. Fully-covered metallic stents (FCSEMS) are swiftly efficient but subject to high migration rates. Because new through-the-scope suturing devices (TTSS) are now available, an alternative to over-the-scope/through-the-scope clips (OTSC/TTSC) or over-the-scope suturing devices (OTSS) can be trialed to anchor FCSEMS and prevent their dislodgement.

**Methods:**

We performed a preclinical comparison on a porcine model of the ability to prevent FCSEMS migration with TTSS, OTSC, and TTSC. Given the promising results, we then performed stent anchoring with TTSS to selected patients with refractory benign strictures. We hereby present these initial procedures and their outcomes.

**Results:**

In preclinical trials, TTSS provided significantly higher resistance to traction than OTSC, TTSC, and no anchoring. We performed eight FCSEMS anchoring with TTSS among six patients, with encouraging technical and clinical outcomes.

**Conclusions:**

This is the first preclinical and clinical description of the benefits of TTSS for FCSEMS anchoring in esophageal refractory benign strictures. Safe and efficient anchoring with TTSS could allow using double-silicon-layered FCSEMS that can be left in place several months for management of refractory benign strictures. This work paves the way for prospective studies assessing FCSEMS anchoring with TTSS.

## Introduction

Benign esophageal strictures are a common occurrence in interventional endoscopy 1 , whose main causes are radiation therapy, esophageal surgery, caustic ingestion, and less frequently peptic strictures, post endoscopic submucosal dissection strictures or structural abnormalities 2 .

The main treatment of these strictures remains endoscopic, with hydrostatic balloon dilation or stepwise Savary bougienage. A refractory stricture has been variously described, among other definitions, as the persistence of dysphagia despite five dilations at 2-week intervals and/or inability to obtain an esophageal diameter of 14 mm or more 3 4 , whereas a recurrent stricture can be defined as inability to maintain this diameter for at least 4 weeks. Management of refractory or recurrent strictures relies on corticosteroid injections or radial incisions alone or combined with dilations, all of which have some limitations 5 . Fully-covered self-expendable metallic stents (FCSEMS) are another therapeutic option for refractory or recurrent strictures 6 which, despite a strong and immediate efficacy in alleviating dysphagia 7 , have poor overall results mostly due to migration rates of up to 30% 8 and stricture recurrence rates of roughly 50%. Epithelial hyperplasia along the metal mesh does not allow standard esophageal FCSEMS to be left in place for more than 4 to 6 weeks 9 . Esophageal stents covered internally and externally with a double silicon layer (DSL-FCSEMS) can be left in place for more than 6 months but their even higher migration rate precludes their use in most patients 10 . Anchoring stents to the esophageal mucosa by using through-the-scope clips (TTSC) has not proven to be reliable, but over-the-scope suturing devices (OTSS) or over-the-scope clips (OTSC) have shown some efficacy, although they are sometimes difficult to apply (OTSS) or demanding to remove (OTSC) 6 11 12 .

Recently, a newly developed through the scope suturing (TTSS) device has been made available, which could be a promising alternative to anchor esophageal stents 13 .

We believe that the combination of TTSS (quicker and easier to manipulate than OTSC/OTSS) with DSL-FCSEMS could be a promising approach for treatment of refractory or recurrent benign strictures.

To investigate the potential benefits of this combination, we performed a preclinical study on porcine ex-vivo models to compare resistance to traction of different anchoring systems (OTSC, TTSC, TTSS) with a DSL-FCSEMS. Some of our patients with refractory benign strictures that had reached therapeutic dead ends were then selected for third-line therapy with esophageal stenting with anchoring of the stent with TTSS.

We present in this publication our first clinical cases supported by preclinical data.

## Methods

### Preclinical trial

**Fig. 1:**
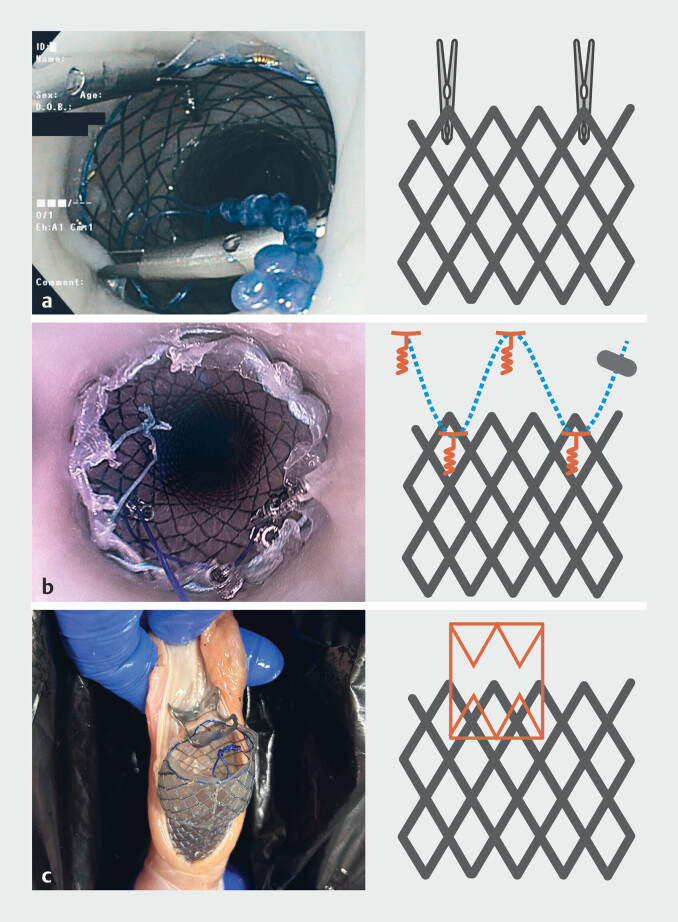
Different anchoring strategies tested on the porcine ex-vivo model. **a** TTSC: Two clips placed at opposite sides of the stent, biting the metal mesh and the adjacent mucosa. **b** TTSS: Tacks 1 and 3 are screwed above the stent and tacks 2 and 4 through the silicon layer of the stent with a suture cinch securing the thread. **c** OTSC Biting the stent and the adjacent

Porcine stomachs were positioned in a dedicated box with their esophagus attached to introduce the endoscope (EASIE-R model 14 ). A DSL-FCSEMS (EBN, Hanarostent, MI-Tech, South-Korea) was positioned under endoscopic control across a mildly knot-created stricture in the mid-esophagus. The stricture was created by tightening the esophagus around the endoscope, allowing the stricture to be crossed with a slight give. This approach ensures reproducibility of the degree of stricture between measurements and across models. The stricture was located in the mid-esophagus to allow complete stent coverage without subcardial protrusion. The proximal part of the stent was anchored to the esophagus with different techniques. Fig. 1 recapitulates the different fixation methods used, as described here.

In TTSC, two clips (17 mm, Resolution 360, Boston Scientific, United States) were positioned endoscopically to anchor the stent or four tacks of the device (X-Tack, Boston Scientific, United States) were positioned to anchor the stent. The first tack was positioned above the stent. The screwing system allows the tack to get anchored up to the muscularis layer. The second tack was positioned through the silicon layer, between the mesh of the stent. In order to penetrate the muscle, we added several manual rotations to the stent after screwing. For the four-tack procedure, the third tack was again positioned above the stent and the fourth through the stent. A suture cinch (Boston Scientific) was positioned at the end to close the suture.

In OTSC, an 11-mm clip (OVESCO, Germany) was positioned endoscopically to bite the upper part of the stent and the esophageal wall above.

**Fig. 2:**
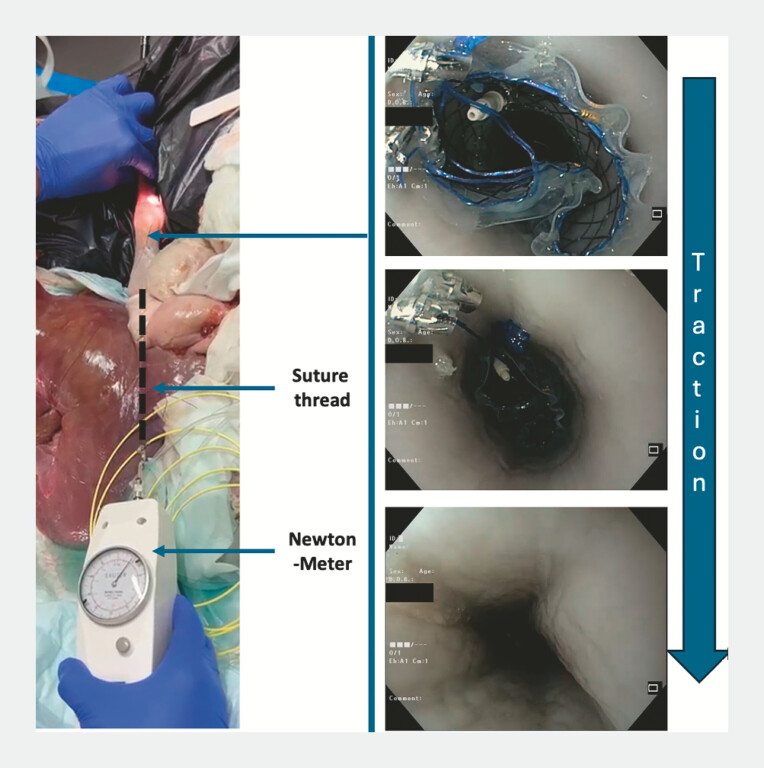
Ex-vivo porcine model. Suture thread attached to the distal part of the esophageal stent. Traction is applied and measured with the newton-meter allowing to determine the force required to dislodge the stent.

To compare these different techniques, we attached a suture thread to the distal part of the stent ( Fig. 2 ). This thread was caught outside of the stomach through a small incision in the anterior gastric body. Traction could thus be applied following the esophagus axis. We measured the traction force in Newton (N) required to dislodge the stent from its position. The traction force was measured with an analogic Newton-meter (Sauter, Switzerland). We calculated that three measures were enough to evidence a 50% difference between TTSS/OTSC and TTSC/anchoring (α = 5%/β = 20%). The significance of the measured differences between TTSS, TTSC, OTSC, and no anchoring was assessed using ANOVA (R-Software-V4.4.2).

### Clinical cases

Patients were included in a single tertiary center (Beaujon Hospital, AP-HP, Clichy, France) from November 2024 to June 2025. Procedures were performed by three senior endoscopists.

Our first treated patient presented with a postsurgical fistula and failure of coverage by a FCSEMS due to stent migration. Subsequently, we reserved this treatment for patients with refractory or recurrent benign strictures.

Refractory strictures were defined as persistence of dysphagia despite five dilations performed at 2-week intervals, achieving or exceeding a diameter of 14 mm, and/or inability to achieve this diameter despite multiple dilations. A recurrent stricture was defined as inability to maintain the achieved diameter for more than 4 weeks 3 4 15 . Patients with refractory or recurrent strictures were, if possible, treated with “second-line therapies” such as radial incisions, stent placement, or corticosteroid injections. If these measures remained ineffective, we performed FCSEMS placement and anchoring with the X-Tack system. If effective and well tolerated, the stent was replaced at 6 weeks by a DSL-FCSEMS anchored with X-Tacks to be kept for several months.

We present in our results the description of our first cases and their preliminary outcomes. Data were collected retrospectively after ensuring patient non-opposition.

## Results

### Preclinical trials

**Fig. 3:**
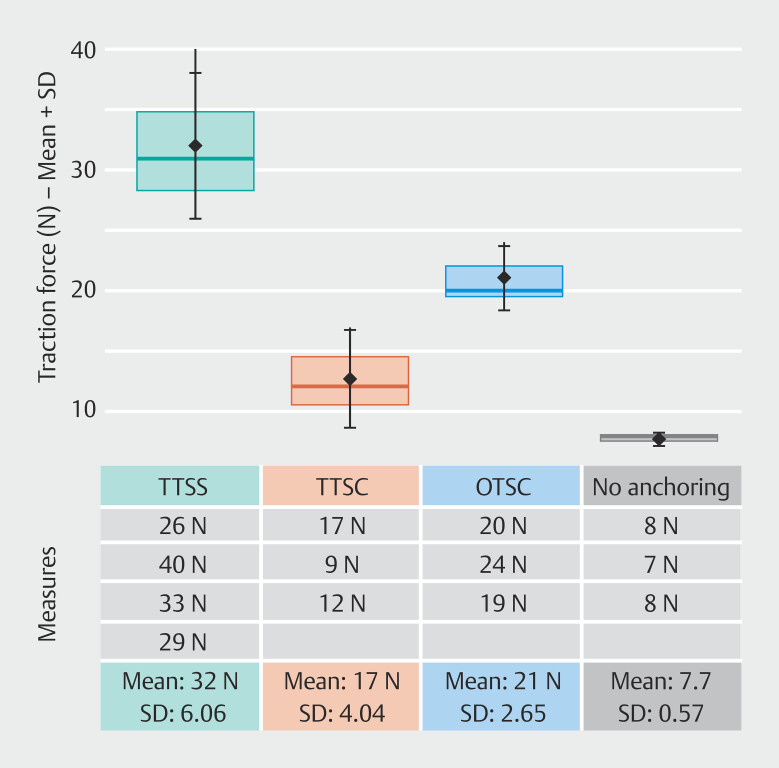
Traction force necessary to dislodge the esophageal stent.

We performed four X-Tack fixations and three TTS/OTSC fixations on our model. We completed with three traction tests with no anchoring. Measurements are displayed in Fig. 3 . Mean traction force required to dislodge the stent with TTSS, TTSC, OTSC, and no anchoring, were 32N, 12.7N, 21N, and 7.7N, respectively. The ANOVA highlighted a significant difference between the groups ( *P* = 0.00015). Post-hoc Tukey’s test evidenced a difference between TTSS and TTSC ( *P* = 0.003), between TTSS and no anchoring ( *P* = 0.0001), between TTSS and OTSC ( *P* = 0.03) and between OTSC and no anchoring ( *P* = 0.02). There was no significant difference between OTSC/TTSC ( *P* = 0.5) and TTSC/no anchoring ( *P* = 0.13).

As for human applications, positioning of the TTSC and OTSC was not easy. Two of the TTSCs available for the procedures were mis-positioned, requiring repeat of the procedure. TTSS was quite easy with a 100% technical success rate. With a trained assistant, anchoring took 5 to 7 minutes with TTSS, including installation of the device.

Stent dislodgement induced dilaceration of the mucosa with small perforations with the TTSC, TTSS, and OTSC. We noted a transmural penetration for two of the tacks through the pig esophagus.

### Clinical cases

Anchoring of an esophageal stent with the XTack device.Video 1

We performed a total of eight FCSEMS fixations with the X-Tack system in six patients. Patient characteristics, stenting indications, prior therapies, procedure descriptions, and outcomes are detailed in Table 1 . Outcomes were last assessed on January 7, 2025. Video 1 shows the anchoring procedure.

**Table:** **Table 1** Clinical cases of stent anchoring with XTack.

Patient characteristics	Stenting indication	Previous therapies	Procedure description	Outcomes
**Patient 1** 83-year-old male Medical history: Cholecystectomy Esophageal adenocarcinoma treated with radiotherapy in 2022 with local relapse motivating surgical resection with Lewis-Santy esophagectomy	Disruption of esophageal continuity over 15 mm at the esophagogastric anastomosis (30 cm from the dental arches). High-output fistula between the esophagus and a large pleural collection.	Failure of double pigtail stents Failure of VAC-Therapy with ESO-Sponge Migration of a first coverage FCSEMS.	**Stent anchoring:** Positioning of a FCSEMS of 22 mm x 140 mm across the loss of esophageal continuity Anchoring with XTack following the “W” pattern. Total procedure duration: 46 min	**Technical success** . No residual leak under fluoroscopy **No complication** **No clinical success** , persistence of a high output fistula despite covering by the FCSEMS Removal of the stent scheduled 1 month later
			**Stent removal:** The stent and XTack were still in place 1 month after their positioning Removal with a foreign body forceps of the Cinch and of the stent.	**Technical success** **No complication** related to the stent or its removal. **No clinical success** , the patient kept a high-output fistula despite all the attempted treatments and died 3 months later.
**Patient 2** 72-year-old male Medical history: Surgical prostatectomy Esophageal adenocarcinoma treated with Lewis-Santy esophagectomy.	Refractory anastomotic stricture Short anastomotic stricture (< 1 cm) located at 32 cm from the dental arches	Multiple dilations with a maximum diameter of 18mm, some followed by medically or endoscopically-treated perforations Radial incisions Corticosteroids injection Several FCSEMS that either migrated within few weeks or had to be removed at 1 month with early dysphagia relapse	**Stent anchoring no. 1** Large radial incision of the stricture covered by a FCSEMS of 22 mm x 120 mm Anchoring with XTack following the “W” pattern Total procedure duration: 1h46min	**Technical success** **No complication** Clinical success with possibility to eat almost normally Removal scheduled 6 weeks later.
			**Stent removal no. 1** The stent and XTack were in position 6 weeks after their positioning Removal with a foreign body forceps of the Cinch and of the stent. One tack was still anchored in the esophagus and was easily removed with the forceps. The stricture being completely calibrated, no additional stent is positioned	**Technical success** **No complication** related to the stent or its removal. Clinically: Dysphagia relapse 3 weeks after stent removal Patient scheduled to insert another FCSEMS anchored with XTack
			**Stent anchoring no. 2** Positioning of a 22mm x 120 mm FCSEMS Anchoring with XTack following the “W” pattern. Error in securing one of the tacks to the device, leading to its disunion. Anchoring performed with 3 of the 4 tacks. Total procedure duration: 1h 40	**Technical difficulties** with partial anchoring of the stent **No complication** **Clinically** : Swift clinical improvement but migration of the stent 3 weeks after its positioning inducing a relapse of the dysphagia. Patient scheduled for stent removal and replacement with a long-lasting DSL-FCSEMS anchored with XTack
			**Stent anchoring no. 3** Stent migrated under the stricture with no remaining Tack in the mucosa. Dilation of the stricture and removal of the migrated stent with a forceps. Positioning of a DSL-FCSEMS to be left in place for 6 months and anchoring with XTack following the “W” pattern. Total procedure duration: 1h 10 min	**Technical success** **No complication** Clinical success: Complete dysphagia relapse. At the date of publication (4 months after stent anchoring), the patient is still improved with no sign of migration.
**Patient 3** 61-year-old male Medical history: Frontal lobe disorder Depression Caustic ingestion	Refractory caustic stricture Long stricture (5 cm) starting at 31 cm from the dental arches.	Multiple Savary dilations up to 15 mm without sustained improvement Esophageal FCSEMS that migrated after only a few days without clinical improvement.	**Stent anchoring** Positioning of a 22 × 120 mm FCSEMS with antireflux valve after difficult crossing of a very tight stricture with a hydrophile wire. Anchoring with X-TACK following the “W” pattern Total procedure duration: 1h 40	**Technical success** **No complication** **No clinical success** : Persistent aphagia despite complete esophageal permeability with the stent. Probable esophageal dysfunction associated with the stricture following caustic ingestion. Removal of the stent scheduled 1 month after to prevent its incarceration
			**Stent removal** The stent and XTack were in position 1 month after their positioning Removal with a foreign body forceps of the cinch and of the stent. No remaining tack after stent removal. Regarding the poor clinical outcome, no DSL-FCSEMS was positioned.	**Technical success** **No complication** related to the stent or its removal. **Clinically** : Persistent aphagia. Patient scheduled for surgery.
**Patient 4** 67-year-old male Medical history: Diabetes Hypertension Esophageal adenocarcinoma treated with Lewis-Santy esophagectomy.	Refractory anastomotic stricture Short anastomotic stricture (< 1 cm) at 23 cm from the dental arches.	Multiple hydrostatic and Savary dilations up to 18 mm Esophageal FCSEMS that migrated after only a few days without clinical improvement.	**Stent anchoring** Positioning of a 22 × 120 mm FCSEMS with anti-reflux valve. Proximal part of the stent reaching the cervical esophagus. Difficulties to anchor the stent with misplacement of one of the Tacks. Regarding the lack of available space above the stent. The three remnant tacks are screwed through the stent. Total procedure duration: 1h 04	**Technical difficulties** with partial anchoring of the stent with 3 of the 4 tacks **No complication** **Clinically** : Swift clinical improvement but migration of the stent 4 weeks after its positioning requiring its removal. Patient currently undergoes iterative dilations and radial incisions/corticoid injections with mild clinical improvement.
**Patient 5** 44-year-old male Medical history: Depression Peritonitis and several intensive care complications following caustic ingestion	Refractory caustic stricture Long stricture (5 cm) ranging from 30 to 35 cm from the dental arches.	Multiple Savary dilations up to 18 mm without sustained improvement	**Stent anchoring** Positioning of a 22 × 120 mm DSL-FCSEMS covering the stricture Anchoring with X-TACK following the “W” pattern Total procedure duration: 1h 01	**Technical success** **No complication** **Intermediate clinical success** : Improvement of the dysphagia but stent-related pain and reflux motivating an early removal of the stent.
			**Stent removal** The stent and XTack were in position 4 weeks after their positioning Removal with a foreign body forceps of the Cinch and of the stent. No remaining tack after stent removal. The stricture was completely calibrated	**Technical success** **No complication** **Clinical success** . No relapse of the dysphagia at the date of publication and improvement of the patient’s symptoms.
**Patient 6** 64-year-old male Medical history: None Caustic ingestion treated with gastrectomy and esophagojejunostomy	Refractory anastomotic stricture Long stricture (8 cm) ranging from 28 to 36 cm from the dental arches.	Multiple Savary dilations up to 18 mm without sustained improvement	**Stent anchoring** Positioning of a 22 × 140 mm DSL-FCSEMS covering the stricture Anchoring with X-TACK following the “W” pattern Total procedure duration: 55 min	**Technical success** **No complication** Clinical success. No relapse of the dysphagia and no stent migration at the date of publication
DSL, double silicone layer; FCSEMS, fully-covered self-expanding metal stent.				

Regarding patient characteristics and indications, all patients were male, with a median age of 65.5 years (range 61–72). One patient had an anastomotic fistula, three had refractory anastomotic strictures, and two had refractory strictures following caustic ingestion. All strictures were refractory despite multiple dilations (up to at least 15–18 mm).

Regarding the procedures, five patients received standard FCSEMS with a 22-mm diameter and three received DSL-FCSEMS. Stent removal, when necessary, was straightforward and consisted of simply pulling the cinch with foreign-body forceps. Most of the tacks were dislodged upon stent removal; any remaining tacks could be easily retrieved one by one with forceps. Median procedure duration (from entry to exit of the operating room) was 67 minutes (range 58 minutes to 1 hour, 40 minutes).

Regarding technical success, two of the eight anchoring procedures were technically imperfect due to mispositioning of one of the four tacks. No adverse events were noted during anchoring or stent removal. Two stents migrated before their scheduled removal, 3 and 4 weeks after their anchoring, respectively. Both were standard FCSEMS; no migration occurred with DSL-FCSEMS.

Regarding clinical success, one patient had a persistent leak despite correct stent positioning, one remained improved and required no further procedures 4 months after the last stent placement, one developed functional aphagia despite proper stricture coverage, and one experienced early stent migration and returned to an iterative dilation program. One patient had the stent removed after 1 month due to intolerance but remained improved by the stricture calibration, and one showed initial improvement but had only short follow-up at the time of publication.

## Discussion

TTSS devices have only been made available recently. Most of the few published series included fistula/perforations repair cases 16 . One single publication described use of TTSS devices to coaxially suture two stents to one another in order to create a longer stent covering large gastrointestinal defects 17 and one case report described esophageal stent fixation with a helix tack 13 . To our knowledge our work is the first preclinical evaluation and clinical case series of TTSS use for stent anchoring in treatment of non-tumoral refractory esophageal strictures. Although our clinical experience is obviously limited, the eight instances of TTSS anchoring tend to demonstrate technical feasibility, including for double silicone-layered stents, with neither major technical hurdle nor special skill requirement.

To our knowledge, this is also the first publication describing a preclinical comparison of different stent anchoring methods. The combination of the EASIE-R model with tensile strength measurement allowed us to compare TTSS, TTSC, OTSC, and no anchoring. We highlighted a significantly higher resistance to traction with TTSS compared with no anchoring with a ratio of 4.15/1, but also to TTSC (ratio of 2.5/1) and even to OTSC (ratio of 1.5/1) with significant differences despite the limited data available.

TTSS appeared to be quick and efficient. With a trained helper to manipulate the screwing handle, anchoring can be achieved within 5 minutes. Contrary to OTSC, removing the stent is easily done by simply cutting the thread with endoscopic scissors or by pulling the cinch with a foreign-body forceps. We experienced a few transmural penetrations of the tacks in the preclinical models. The esophagus wall in 35- to 40-kg pigs has a mean thickness of 3.6 mm 13 , slightly less than human adults (4.4 to 5.3 mm) 18 19 . X-Tacks have a 3.5-mm long screwing system allowing them to reach the muscular layer without breaching it. Initial human cases performed for defect closing or stent anchoring have not reported similar complications 20 21 22 23 24 25 . Although this model is cost-effective and reproducible, it is an oversimplification of the in vivo environment due to absence of peristalsis and cannot be used alone to compare anchoring techniques.

We report our first cases of patients undergoing TTSS stent anchoring for refractory esophageal strictures and one particular case of stent anchoring to cover a large fistula. We experienced two technical failures related to an equipment handling error, inducing loss of one of the four tacks with an imperfect anchoring. In both cases, stent migration occurred. In all of the other cases, the stent remained in place until its scheduled removal or at the date of publication. DSL-FCSEMS are long-lasting but prone to migration 10 . There was no migration of any DSL-FCSEMS when properly anchored with X-Tack. Regarding clinical outcomes, one patient achieved sustained clinical success and is currently free of dysphagia 4 months after DSL-FCSEMS placement, despite a history of multiple stent migrations. Two other patients showed improvement but had limited follow-up. Two patients did not experience clinical improvement (one with a fistula, one with dysphagia) despite correct stent positioning and patency. One patient had early migration without any clinical benefit from stenting. Despite the short follow-up period and the retrospective nature of this series, these results in patients facing therapeutic dead ends are encouraging.

In light of this early experience, several tips can be proposed. For anchoring to properly work, the tacks have to drill through the silicon and reach the muscular layer of the esophagus. The device, therefore, must be as perpendicular as possible to the esophageal wall and pressure must be applied while screwing with several extra turns of the tack to cross the silicon layer.

In one of our cases, lack of available space above the stent forced us to perform a different fixation pattern with all the tacks through the stent. This strategy is also advised if partially-covered SEMS are positioned because the tacks can easily penetrate through the uncovered parts of the stent. In case of difficulties in positioning the tacks through the mesh of the stent, we propose an alternative option with screwing the four tacks above the esophageal stent, creating an anchor line with the thread and then using TTSC to clip the thread to the stent. Indeed, TTSC weakness lies in the poor anchorage possibilities on the esophageal wall. This last possibility will be further evaluated on our preclinical model before human application.

**Fig. 4:**
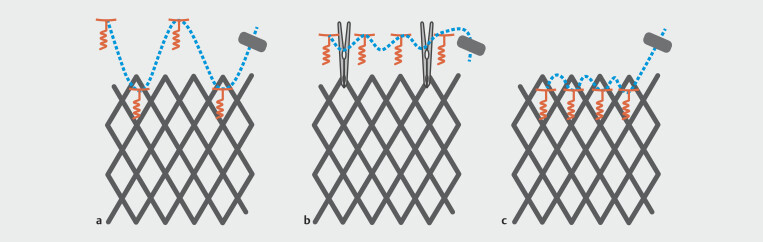
Different anchoring patterns with TTSS. **a** “W” Pattern. Tacks 1 and 3 are screwed above the stent and tacks 2 and 4 through the silicon layer of the stent with a suture cinch to secure the thread. The four tacks distribute the tensile force. **b** The four tacks are screwed above the stent. The thread is secured with a cinch and two TTSC are used to join the stent to this anchoring line. **c** For partially covered stents or if no space is available, the four tacks are positioned through the stent.

The different anchoring pattern with X-Tacks are described in Fig. 4 .

Stents are immediately efficient for symptom relief in refractory strictures but are often relegated to treatment of acute fistulae and post-dilation complications because of high migration rates. Anchoring stents to the esophageal wall may be a solution to overcome this shortcoming and has already been investigated. OTSC or OTSS have been shown to significantly reduce migration rates compared with no anchoring 11 or to TTSC 26 .

However, OTSS is a cumbersome and expensive procedure requiring specific training, a complex set-up on the endoscope, and is difficult to handle inside the esophagus. OTSCs can also be difficult to use because they have to bite simultaneously on the stent and above, thus requiring perpendicular positioning inside the esophagus. Whenever properly positioned, OTSC cannot be easily removed for stent exchanges without using a specific device to release the clip. TTSC are equally challenging to position in the esophagus and a recent meta-analysis showed almost no difference between TTSC and no anchoring at all 26 .

We believe that using long-lasting esophageal stenting with double silicone-layered FCSEMS combined with TTSS has potential to improve management of refractory benign strictures and seems technically easier and possibly more reliable than previously existing anchoring devices.

The conclusions of this work are subject to several limitations. This was a single-center study including the first patients treated at our institution for this indication, which implies a learning curve and accounts for some of the technical difficulties encountered. Refractory strictures remain relatively uncommon, even in expert centers, and this small retrospective series can only serve to demonstrate proof of concept and procedure feasibility. This work will be followed by a prospective study to assess clinical efficacy and safety outcomes.

## Conclusions

We provide the results of combined preclinical and clinical work investigating TTSS for FCSEMS anchoring to prevent their migration in treatment of benign refractory strictures. Our preclinical results highlight significantly higher resistance to traction of the TTSS compared with TTSC and OTSC and our first clinical cases suggest technical feasibility and pave the way for prospective studies to determine whether this new method will result in clinical benefit.
